# Zoonotic Potential of Influenza A Viruses: A Comprehensive Overview

**DOI:** 10.3390/v10090497

**Published:** 2018-09-13

**Authors:** Ahmed Mostafa, Elsayed M. Abdelwhab, Thomas C. Mettenleiter, Stephan Pleschka

**Affiliations:** 1 Institute of Medical Virology, Justus Liebig University Giessen, Schubertstrasse 81, 35392 Giessen, Germany; Ahmed_elsayed@daad-alumni.de; 2 Center of Scientific Excellence for Influenza Viruses, National Research Centre (NRC), Giza 12622, Egypt; 3 Institute of Molecular Virology and Cell Biology, Friedrich-Loeffler-Institut, Federal Research Institute for Animal Health, Südufer 10, 17493 Greifswald-Insel Riems, Germany; Sayed.Abdel-Whab@fli.de (E.M.A.); thomas.mettenleiter@fli.de (T.C.M.)

**Keywords:** Influenza A virus, zoonosis, pathogenicity, evolution, pandemic

## Abstract

Influenza A viruses (IAVs) possess a great zoonotic potential as they are able to infect different avian and mammalian animal hosts, from which they can be transmitted to humans. This is based on the ability of IAV to gradually change their genome by mutation or even reassemble their genome segments during co-infection of the host cell with different IAV strains, resulting in a high genetic diversity. Variants of circulating or newly emerging IAVs continue to trigger global health threats annually for both humans and animals. Here, we provide an introduction on IAVs, highlighting the mechanisms of viral evolution, the host spectrum, and the animal/human interface. Pathogenicity determinants of IAVs in mammals, with special emphasis on newly emerging IAVs with pandemic potential, are discussed. Finally, an overview is provided on various approaches for the prevention of human IAV infections.

## 1. Introduction

Influenza is a contagious respiratory disease caused by influenza viruses (IVs). IVs are categorized antigenically based on the variations of the nucleoprotein (NP) into four genera: influenza A viruses (IAV), influenza B viruses (IBV), influenza C viruses (ICV), and influenza D viruses (IDV) [1]. Together with Isavirus, Thogotovirus, and Quaranjavirus, they compose the family *Orthomyxoviridae*. Both human IAV and IBV trigger seasonal global epidemics, while human infections with ICV are less frequent and generally cause mild illness without reported epidemics. IDV mostly infect cattle and are not yet known to infect humans [2].

**Figure 1:**
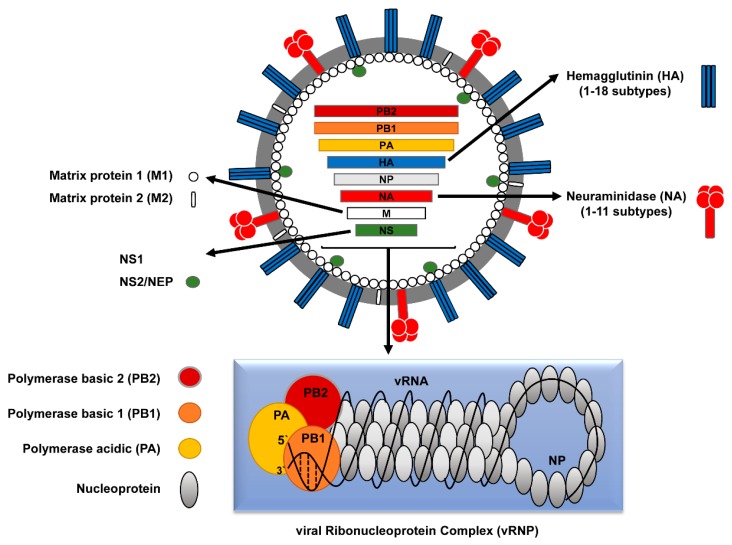
Schematic structure of influenza A virus (IAV). The envelope of the IAV particle, which is derived from the host cell plasma membrane, contains three trans-membrane proteins; two surface glycoproteins designated as hemagglutinin (HA) and neuraminidase (NA) and the proton channel matrix protein 2 (M2). The matrix protein 1 (M1) underlies the inner surface of the viral envelope and associates with NEP and viral ribonucleoprotein complexes (vRNPs). The eight vRNPs comprise eight negative-strand RNA segments associated with the nucleoprotein (NP) and three RdRp polymerase subunits (PA, PB1, PB2).

IVs have a segmented, negative sense, single-stranded (-ss) viral RNA (vRNA) genome. Both IAV and IBV contain eight vRNA segments, while ICV and IDV contain only seven segments [3]. The eight genomic segments of IAV encode at least 10 viral proteins (Figure 1). 

Each segment encodes at least one structural protein: polymerase basic protein 2 (PB2, segment 1), polymerase basic protein 1 (PB1, segment 2), polymerase acidic protein (PA, segment 3), hemagglutinin (HA, segment 4), nucleoprotein (NP, segment 5), neuraminidase (NA, segment 6), matrix proteins (M1 and M2, segment 7), and non-structural proteins (NS1 and NS2 or nuclear export protein (NEP), segment 8) (Table 1).

The viral envelope contains two viral transmembrane glycoproteins; hemagglutinin (HA, ~80%) and neuraminidase (NA, ~17%). The HA protein forms a trimer, which protrudes approximately 13.5 nm from the viral surface and has a rod-like shape, while the NA protein forms a tetramer with mushroom-like shape [3,4,5]. In addition, the IAV envelope contains a small number of an integral tetrameric membrane protein called Matrix protein 2 (M2) with ion channel activity [6]. The Matrix protein 1 (M1) underlies the inner surface of the envelope. Furthermore, the NEP/NS2 protein is detected in minor amounts and is a consistent component of influenza virions [7]. The viral core comprises the eight vRNA segments (890–2341 nucleotides (nt)) in association with the viral nucleoprotein (NP) and the RNA-dependent RNA-polymerase (RdRp) subunits (PB2, PB1, and PA) to constitute the biological active replication/transcription units of influenza virus, called viral ribonucleoprotein (vRNP) complexes [3] (Figure 1 and Table 1).

**Figure 2:**
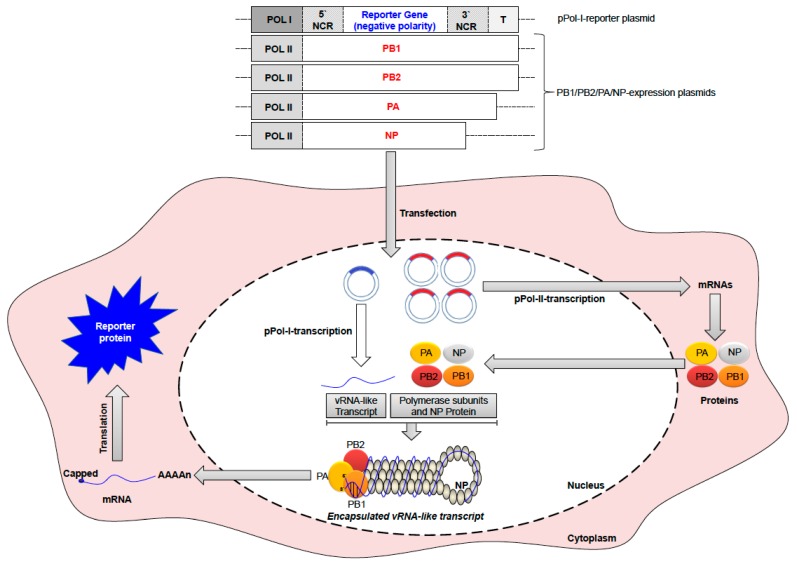
Overview of minigenome assay for IAV. The DNA of four expression plasmids, encoding the PB1, PB2, PA, and NP of IAV under control of polymerase II promoter (Pol-II), are co-transfected into host cell with reporter plasmid, which is carrying the ORF of the reporter gene (e.g., Luciferase, CAT, GFP) in a negative polarity and flanked by the NCRs of an IAV segment. Transcription is controlled by a Pol-I promoter (Pol-I) and a Pol-I terminator (T) to express a specific vRNA-like Pol-I-transcript of the reporter gene. The expression of the viral proteins together with the vRNA-like reporter gene transcript results in the in vitro reconstitution of RNP complexes. The RNP complexes generate the corresponding mRNA, which is then translated into the reporter protein of unique enzymatic, fluorescent or chemiluminescence activity. The represented regions in plasmid constructs are not drawn to scale.

As a complex genomic entity with unique structure and function among RNA viruses, the vRNP of IAVs is composed of the RNA-dependent RNA polymerase (RdRp) subunits (PB2, PB1 and PA) as a globular head linked to rod-shaped structures of the vRNAs folded on the NP protein. A breakthrough technology that allowed detailed comprehension of the vRNP structure/function, to deduce the mechanisms of RNA replication and transcription, the intracellular trafficking of the viral genome, selective packaging of the vRNPs and viral gene reassortment, was the development of the in vitro reconstitution of recombinant vRNPs that represent efficient replicons. This gave also rise to the development of minigenome assays and different reverse genetic systems for IAV to study the impact of specific adaptive genetic changes and viral segments reassortment of zoonotic potential [36,37,38] (Figure 2).

## 2. Classification of Influenza A Viruses (IAVs)

Unlike influenza B, C, and D viruses, IAV are subtyped according to the antigenicity of the surface glycoproteins into 16 HA- and 9 NA-subtypes. Except for bat-origin influenza-like viruses H17N10 and H18N11, all IAV subtypes were isolated initially from avian hosts (Table 2) [39,40,41,42,43,44].

### 2.1. Human versus Avian IAVs

Humans are susceptible to infection with influenza A, B, and C viruses. A limited number of IAV subtypes are circulating in humans such as the H1N1 and H3N2 subtypes leading to seasonal infections or occasional pandemics [45,46,47,48]. Other IAV subtypes can occasionally cross the species barrier from birds to mammals/humans such as H5N1, H5N6, H6N1, H7N2, H7N3, H7N4, H7N7, H7N9, H9N2, H10N7, and H10N8, causing sporadic infections and/or fatalities [49,50,51,52,53,54,55]. However, almost all subtypes of IAV (except H18N11 and H17N10) are classified as avian influenza viruses (AIV). Aquatic birds are the main natural reservoir of AIV [43,56].

### 2.2. Highly versus Low Pathogenic Avian Influenza Viruses (HPAIV vs. LPAIV)

AIV are typed according to their pathogenicity in chickens into low pathogenic (LP) and highly pathogenic (HP) strains. LPAIVs are maintained in wild aquatic birds almost without developing severe clinical signs of the disease. The clinical signs in domestic poultry induced by LPAIVs include a body weight reduction and/or a slight drop in egg production in layers poultry [57]. In contrast to LPAIV, the HPAIV phenotype is restricted to H5Nx, H7Nx, and H9N2 subtypes that carry a multibasic cleavage site in their HA protein [58,59] and cause up to 100% mortality in several bird species [57,60,61]. Until the mid-1950s, all HPAIVs were characterized as H7-subtypes, while, in 1959, the first outbreak with HPAIV H5N1 in chickens was reported [62]. Except for the fatal outbreak in terns caused by HPAIV H5N3 in South Africa, the HPAIV outbreaks were only reported in flocks of domestic birds until 2002 [63]. Since that time, HPAIVs were frequently reported to cause fatal outbreaks in wild aquatic and terrestrial birds [64,65,66,67].

## 3. Evolution and Epidemiology of IAV

**Figure 3:**
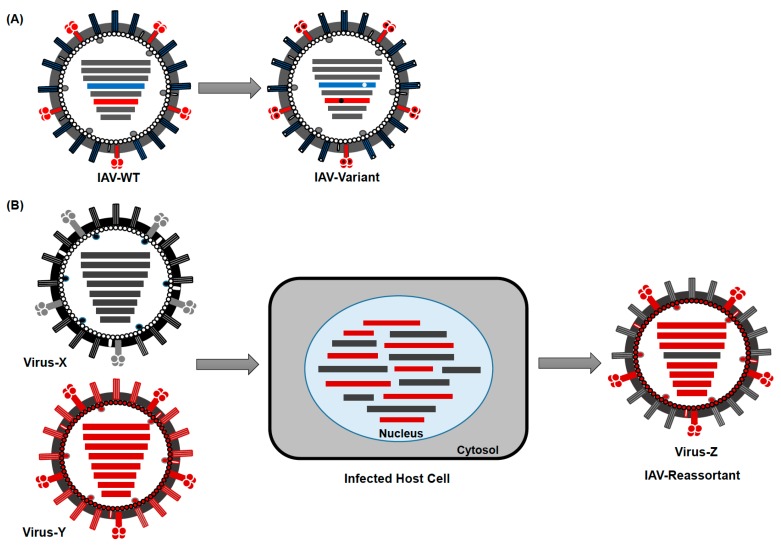
Evolution mechanisms of IAV. (**A**) Antigenic Drift: Gradual accumulation of mutations in the genome of IAVs leads to emergence of new virus variants. Mutations in the HA (blue) and NA (red) can affect the antigenic epitopes leading to antigenically new variants. (**B**) Antigenic Shift: The exchange/reassortment of genetic segments between two or more invading IAVs in a host cell can lead to emergence of (antigenically) distinct new subtype(s).

IAV evolve mainly by two mechanisms: (1) through accumulation of point mutations due to the lack of a proof-reading function of the RdRp, leading to aa changes (referred to as antigenic drift) (Figure 3A) and (2) by reassortment of viral segments from different IAV during co-infection (referred to as antigenic shift) (Figure 3B). Interestingly, the point mutation rate is higher in human than in avian IAV [71]. In addition, a slower evolution rate has been observed in IAV isolated from wild aquatic birds compared with those from terrestrial poultry, swine or humans. This is probably due to adaptation of IAV to new hosts, while genetic stasis is maintained in its natural reservoir [42,43,72]. Additionally, reassortment was only reported to take place within each influenza virus genus (A, B, and C), but has not been observed among different genera [73]. Genetic reassortment and antigenic drift, resulted in 5 documented influenza pandemics since 1900 and in annually repeated seasonal epidemics, respectively [74].

**Figure 4:**
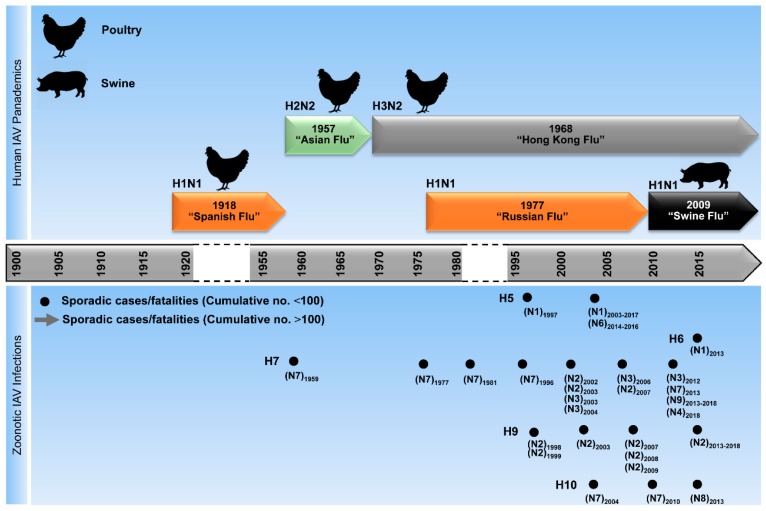
Timeline showing influenza pandemics and epidemics caused by IAVs. The “Spanish Flu” of 1918 was the most devastating influenza pandemic in the 20th century and was likely caused by a zoonotic transmission of an H1N1-type IAV from poultry to humans. This strain disappeared in 1957 when the influenza virus A/H2N2, a reassortant of the H1N1 virus and other avian IAVs, appeared and led to the second influenza pandemic—the “Asian Flu.” In 1968, H3N2, a novel reassortant strain between the H2N2-type and an H3-type virus, displaced the H2N2 strain in the human population and led to the “Hong Kong Flu”—the third influenza pandemic. In 1977 the H1N1 strain reemerged, resulting in the “Russian Flu”. In 2009, a new H1N1 reassortant was transmitted from swine to humans leading to the first pandemic of the 21st century—the “Swine Flu.” In parallel, different avian influenza A virus strains (H5-, H6-, H7-, H9-, and H10-types) have occasionally crossed the host barriers causing mild-to-fatal infections in humans.

Unlike epidemics, pandemics (Figure 4) can spread over a wide geographic area in relatively short time resulting in thousands or even millions of fatal infections. The notorious Spanish influenza (H1N1) led to the most dramatic pandemic of the last century, globally killing more than 25 million people in 25 weeks between 1918/1919. Subsequently, a new pandemic strain—Asian Flu (H2N2)—a reassortant of 1918/H1N1 and the HA/NA/PB1 segments of an AIV, emerged in China in 1957 leading to at least one million fatalities. In 1968, the pandemic Hong Kong Flu (H3N2), a reassortant of the 1957/H2N2 and HA/PB1 segments from another avian IAV, emerged to replace the older H2N2 strain and led to about one million deaths [42]. In 1977, Russian influenza, which is thought to be caused by a re-emerged H1N1 virus, spread worldwide, leading to severe infections in humans with a 50% fatality rate among school-aged children [75]. In 2009, a reassortant H1N1 (H1N1pdm09) virus with a unique genome constellation generated in swine led to the first pandemic of the current century, known as “Swine Flu.” The PB2 and PA segments were derived from a North American AIV, the PB1 segment from a human H3N2 virus, the NA and M segments from an Eurasian avian-like swine virus, and the HA, NP, NS segments from the H1N1-type classical swine virus [76,77]. Unlike H2N2, the H3N2 and H1N1 viruses are still circulating in the human population together with IBV strains [78]. In March 2018, a seasonal reassortant IAV-subtype H1N2 with genome segments from seasonal H1N1pdm09 (HA and NS) and H3N2 (PB2, PB1, PA, NP, NA and M) was identified in a 19-months-old patient with influenza-like illness in the Netherlands [79]. However, epidemiological and virological investigation did not reveal additional human infections with this H1N2-subtype in the same region [79].

**Figure 5:**
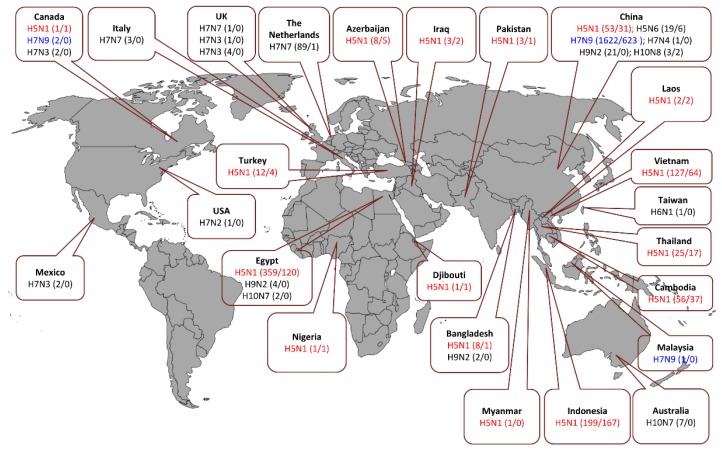
Documented human cases and fatalities caused by zoonotic AIVs. Zoonotic events by H5Nx, H6N1, H7Nx, H9N2, and H10Nx viruses were reported in the indicated countries. In brackets the number of confirmed cases against the number of fatalities until July 2018 countries are indicated [44,91,92,93]. Compared to other human isolates of AIVs (black), H5N1 (red), and H7N9 (blue) demonstrated increased zoonotic potential.

Furthermore, AIVs circulated in different bird species for decades and caused dramatic outbreaks [60]. In 1997, a lethal AIV (type H5N1) was transmitted from chickens to humans, leading to six fatalities out of 18 infected individuals in Hong Kong [51,80,81]. In a second distribution wave in 2003, it was then disseminated via migratory birds throughout Asia and introduced into poultry in Europe, the Middle East, and Africa [82,83]. In parallel, other AIVs, such as H7N2-, H7N3-, H7N7-, H9N2-, and H10N7-type AIV have been occasionally reported to cause mild-to-fatal, sporadic infections in humans in different geographical localities [44,84,85,86,87]. Since 2013, new reassortant AIVs (e.g., H5N6, H6N1, H7N4, H7N7, H7N9, H9N2, H10N8) have crossed the species barrier to infect humans inducing asymptomatic to fatal infections (Figure 5) [49,50,52,55,88,89]. Remarkably, breaking the host barrier was mostly supported by the acquisition of gene segment(s) from other co-circulating AIVs, especially H9N2 [90]. Here we provide a brief description of the genesis of the recent AIVs, which crossed the animal/human interface.

### 3.1. H5N1 (HPAIV)

**Figure 6:**
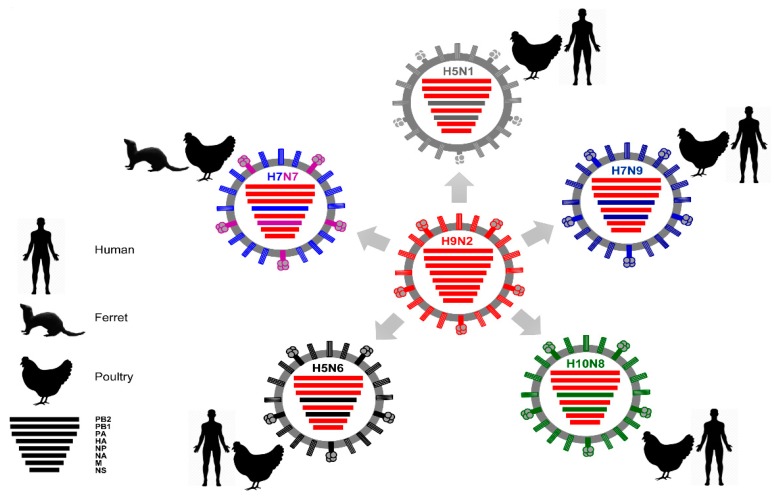
H9N2-type influenza A viruses donate their internal genes to other IAVs. Recent studies revealed the contribution of the internal genes of H9N2 in the genesis of various, recently evolved AIV strains with zoonotic potential [135,138,139]. Poultry (chicken pictogram) served as mixing hosts for emergence of these influenza reassortants, which are then transmitted naturally to humans (human pictogram), or evaluated experimentally in ferrets (ferret pictogram), leading to infections/deaths.

As already noted, the HPAIV H5N1 strain was documented for the first time to have crossed the animal-to-human barrier in 1997, causing zoonotic fatal infections. By genetic analysis of the 1997 HPAIV H5N1, it was revealed that the six internal protein-encoding segments (PB2, PB1, PA, NP, M, NS) were derived from co-circulating avian H9N2 viruses [94,95] (Figure 6). The presence of the same set of segments in two human H9N2 isolates in 1999 indicates an involvement of these segments in interspecies transmission [94]. Continuous evolution of the HPAIV H5N1 viruses that have undergone further multiple reassortments with other AIVs since 2003 was reported in several studies [96,97,98].

From January 2003 to May 2018, 860 human HPAIV H5N1 infections (including 454 fatalities) were reported worldwide. As evidenced by the low incidence rate compared to the H7N9 infections (see Section 3.7, H7N9), the risk of poultry-to-human transmission of HPAIV H5N1 strains is relatively low, but the case fatality rate (53%) is high [91]. Due to their rapid evolution and genetic diversity, HPAIV H5N1 viruses were classified into first order clades (designated clades 0 to 9), which are further diversified into second, third or fourth order clades (e.g., 2.3, 2.3.4, 2.3.4.4) and sub-clades (e.g., 2.3.4.4b) according to their HA segment sequence [99,100,101] and genotypes [102].

In 2003, clade 1 strains were first detected in northern Vietnam. Recently, clade 1 viruses are phylogenetically designated as clades 1.1.1 and 1.1.2 [100,103]. In 2005, clade 2.2 viruses were transmitted by wild birds from Qinghai Lake in western China to other Asian, European and African countries. Clade 2.2 has disappeared worldwide, except for Egypt, where in 2008 the virus became endemic in poultry and evolved into two distinct clades (e.g., 2.2.1.1 and 2.2.1.2) [103,104]. From March 2006 to May 2018, Egypt has reported a total of 359 cases, including 120 deaths mainly caused by these clades [91,104]. Since 2008 viruses evolved in China, Southeast Asia forming the new clade 2.3.2.1, which gained high prevalence, gradually replacing clade 1 viruses, and were also transmitted to Europe. Clade 2.3.2.1 continued its evolution resulting in new, genetically diverse sub-clades (2.3.2.1a, b and c) [103], which are associated with sporadic, but fatal human infections in the last few years [105,106,107,108,109,110]. Currently, clade 2.3.2.1c viruses have become dominant in poultry throughout China, Cambodia, Laos, Indonesia, and Vietnam [103]. Similar to the geographically restricted clade 2.2.1.2 in Egypt, endemic clade 2.1.3 viruses in Indonesia, recently evolved to the new clade 2.1.3.2b [103]. Furthermore, viruses of clade 2.3.2.1c have been introduced to Indonesia, representing a challenge for the implemented strategies for diagnosis and control of HPAIV H5N1 viruses in this area.

Currently, out of the more than 30 previously reported genetic clades, clades 1.1.2 (Cambodia and Vietnam), 2.1.3.2b (Indonesia), 2.2.1.2 (Egypt), 2.3.2.1c (Bangladesh, China, India, Indonesia, Korea, Nepal, and Vietnam), 2.3.4.2 (China), and 7.2 (China and Vietnam) are circulating in domestic poultry [111].

### 3.2. H5N6 (HPAIV)

In April 2014, the first fatal human infection with HPAI H5N6 was reported in China [112]. Patients acquired the infection through contact with infected poultry, particularly in live bird markets. The manifestation of the infection in humans ranged from influenza-like illness including fever and severe pneumonia to death. The genome of this strain was a combination of the HA segment from avian influenza A/H5N2 viruses (clade 2.3.4.4), the NA segment from avian influenza A/H6N6 viruses and internal protein encoding genes from avian influenza A/H5N1 viruses (clade 2.3.2.1c) [112]. To date, three different reassortants of HPAIV H5N6 crossed the species barrier and caused severe infections in human with high mortality rate [113]. The strain “*reassortant A*-HPAIV H5N6” represents the prototypic HPAI H5N6 strain, which led to the first fatal H5N6 infection in humans. Independently, “*reassortant B*-HPAIV H5N6” concurrently resulted from reassortment between H5N8 (clade 2.3.4.4), and H6N6 viruses. Subsequently, the “*reassortant B*-HPAIV H5N6” was subjected to a single reassortment event by which it acquired the six internal genes of a co-circulating influenza A/H9N2 strain resulting in “*reassortant C*-HPAI H5N6” (Figure 6) [113]. In November 2016, a human fatality was associated with the isolation of H5N6 viruses, which originated in poultry after multiple reassortment events of several AIVs, including H3N2, H5Nx, H6N2, H7N3, and/or H9N2 [114,115]. This indicates the high zoonotic potential of these H5N6 viruses. From 2014 to May 2018, a total of 19 laboratory-confirmed human H5N6 infections (including six fatalities, case fatality rate = 32%) in China have been reported to WHO [116].

### 3.3. H6N1 (LPAIV)

Although the avian H6Nx viruses have shown the ability to productively infect and cause illness in mammalian model animals (mice and ferrets) without prior adaptation, the H6-type viruses were not detected in humans until May 2013 [117], when a human infection with LPAIV H6N1 was reported for the first time in Taiwan [118]. Coalescent-based phylogenetic analyses of the human influenza H6N1 strain showed that it was likely to be derived from different H6N1 strains and not by direct reassortment with the co-circulating AIV H5N2 [52]. Due to the high compatibility and the frequent reassortment between H5N2 and the internal genes of H6N1, there is a concern about the indirect contribution of H6N1-internal genes to trigger human infections when incorporated into the genetic backbone of American lineage influenza A/H5N2 viruses [119].

### 3.4. H7N2 and H7N3 (LPAIV and HPAIV)

First evidence of human infections with H7N2 (LPAIV) and H7N3 (LPAIV) was based on positive retrospective serologic analysis of workers involved in the poultry outbreaks in the United States (USA, Virginia) and Italy in 2002 and 2003, respectively [120]. The H7N2 (LPAIV) was then virologically reported in 2003 in an immune-compromised patient in the United States (New York, NY, USA), and later in 2007, it was detected in four human cases in the United kingdom (Wales, UK) [44]. In addition, H7N3 (HPAIV/LPAIV) and H7N3 (LPAIV) led to sporadic human infections in 2004 and 2006 in Canada (British Columbia) and the UK (Norfolk), respectively [120,121]. In 2012, two confirmed mild infections of humans with H7N3 (HPAIV) were detected following exposure to infected poultry in Mexico (Jalisco) [121].

### 3.5. H7N4 (HPAIV)

In February 2018, the Chinese National Health and Family Planning Commission (NHFPC) announced the first non-fatal human infection of H7N4 in Jiangsu Province. The patient was a 68-year-old woman who acquired the infection after exposure to poultry in a live bird market [54].

### 3.6. H7N7 (HPAIV)

Mild human infections with HPAIV H7N7 “conjunctivitis” was reported in 1959, 1977, and 1981 [120]. The most prominent human outbreak of HPAIV H7N7 occurred in the Netherlands in the spring of 2003 leading to 89 human cases including the first reported fatality due to HPAIV H7N7 infection [86,120,122]. The internal genes of this HPAIV H7N7 were of avian origin and were genetically related to the previously circulating LPAIV H7N7 in ducks in the same region in 2000 [86]. In September 2013, three poultry workers in Italy were infected with H7N7 AIV. They acquired the infection after participation in culling of poultry infected with HPAIV H7N7. The patients had conjunctivitis. Genetic analyses of all gene segments indicated high similarity to the H7N7 viruses isolated from chickens on affected farms [123]. In 2013, a previously unrecognized low pathogenic H7N7 lineage carrying the complete set of internal genes from H9N2 subtype AIV was detected in chickens in China and has the ability to infect mammals experimentally (Figure 6) [90].

### 3.7. H7N9 (LPAIV)

In 2013, zoonotic infections with LPAIV H7N9 were first reported in China [49]. The human pathogenic H7N9 is derived from multiple reassortments between AIV H7N9 (NA), H7N7 (HA), and H9N2 (internal proteins coding gene segments) in domestic ducks and chickens [90]. The virus is still maintained in poultry leading only to sporadic human infections. In February 2017, the Chinese province Guangdong reported the first human infection with the mutated trypsin-independent HPAIV H7N9 [124]. Recent studies revealed that the newly emerging HPAIV H7N9 viruses acquired the internal protein-coding genes from co-circulating H9N2 strains [125]. Despite the higher viral polymerase activity, increased replication efficiency and pathogenicity in human, no clear impact on viral transmissibility or virulence was noticed for HPAIV H7N9 [126]. From February 2013 to July 2018, there were a total of 1625 human infections (LPAIV H7N9: 1593 cases, HPAIV H7N9: 32 cases) in China, Malaysia (one case, imported from China) and Canada (two cases, imported from China) with an average of 295 cases per year. The infections caused a total of 623 fatalities (38% case fatality rate) [93,116]. Since 2013, six waves of LPAIV and HPAIV H7N9 were documented (Table 3) [92,127].

As indicated, the highest number of human H7N9 infections has been reported during the fifth epidemic wave, with a geographic range expansion within Chinese provinces [128]. Despite the fact that the outbreaks of LPAIV H7N9 are continually reported in poultry, no new human infections were documented since February 2018. To date, 40 family clusters of 2–3 persons with confirmed or suspected infection with LPAIV and HPAIV H7N9 were reported [116], however, there is no clear evidence that H7N9 virus have evolved to sustain human-to-human transmission.

### 3.8. H9N2 (LPAIV)

Since the mid-1980s, the LPAIV H9N2 circulates extensively worldwide in poultry resulting in high genetic diversity [129,130]. Based on phylogenetic analysis of the HA segment sequence, H9N2 viruses are designated either as Eurasian or American lineages [95,129]. The Eurasian lineage include three genetically distinct sublineages: (1) *Y280-like lineage*, represented by A/duck/Hong Kong/Y280/97 (Y280-like), A/Chicken/Beijing/1/94 (BJ94-like), and A/Chicken/Hong Kong/G9/97 (G9-like); (2) *G1-lineage* represented by A/quail/Hong Kong/G1/97-like (G1-like), and (3) *Korean lineage* represented by A/Duck/Hong Kong/Y439 (Y439-like) and A/chicken/Korea/38349-p96323/96 (Korean-like) [95,131,132].

Since the mid-1990s, the BJ/94-, G9-, and G1-like H9N2 viruses are predominantly circulating in chickens and quails in China. Since 2010, the G1-like lineage demonstrated a widespread distribution and prevalence throughout Asia, the Middle East, North Africa, and Europe [130,133,134].

H9N2 viruses are predominantly isolated from domestic poultry and live-bird markets, which were proven to be risk factors for zoonotic transmission of AIVs from birds to humans [135,136]. The seroprevalence for G9- and G1-like H9N2 antibodies among occupationally exposed populations in Southern China emphasized the high incidence rate of subclinical human infections with both prevalent H9N2 lineages [137]. Moreover, in different geographical locations, H9N2 IAVs have crossed the species barrier due to their mammalian-like characteristics, causing mild to moderate infections [135,138,139]. Globally, since March 2013, a total of 27 laboratory confirmed human clinical infections were reported in three hotspots of human AIV infections (21 cases in China, 4 cases in Egypt, and 2 cases in Bangladesh) (Figure 4) [92]. Currently, the global concern about H9N2 viruses is associated with their ability to donate their genes to other AIV giving rise to high and low pathogenic IAVs that could cross species barriers and infect humans (Figure 6). In addition to the zoonotic H5N6, H7N9 and H10N8 AIV, the H9N2 viruses also donated their internal genes to other IAVs, such as avian H5N1 [140,141], H5N2 [136,141], H1N2, H3N2, H6N2 [141], and H6N6 [142].

### 3.9. H10N7 (LPAIV)

Although outbreaks of H10N7 are uncommon, this virus can sporadically cross the species barrier to mammals including humans. Human infections with H10N7 were occasionally reported from Egypt (2004) and Australia (2010) [143]. Recent H10N7 AIV-associated natural outbreaks in harbor seals and experimental infection of ferrets emphasize that H10N7 may possess a zoonotic potential [144,145,146].

### 3.10. H10N8 (LPAIV)

In late 2013, a fatal human infection with LPAIV H10N8 was identified in China [55]. Notably, the human H10N8 IAV isolate possessed genes coding for internal proteins, which were genetically related to the contemporary AIV H9N2 strains (Figure 6) [50,55], suggesting that this unique genetic constellation was established in poultry. Non-fatal human infections with H10N7 IAV were previously reported in 2004 and 2010 at other geographical localities (reviewed in [44,50]).

## 4. Sources of Human Infections with Zoonotic IAV

Although the infection of humans with zoonotic influenza viruses is less frequent than infections with seasonal influenza viruses, there is a global concern that these zoonotic viruses may acquire mutations in animals or humans that favour the efficient animal-to-human or sustained human-to-human transmission. Poultry and pigs are the major sources of human infections with IAVs. The portal of entry for human infections is mostly through the conjunctiva (e.g., rubbing the eye), nasal and mucosal membranes (e.g., inhalation of dust, droplets), or probably swimming in contaminated pools. Eating of well-cooked meat is not a source of human infections with IAVs until now [147,148,149,150].

Generally, the human infections with AIV were found to be limited to individuals with intensive direct contact with the infected animals and few numbers of family clusters without sustainable human-to-human transmission were reported [151,152,153]. The contact with poultry in live bird markets (LBM) is an important source of human infections with AIV in Asia (e.g., H5N1, H7N9, H9N2, H10N8). In LBM, mixing of different species of birds (chickens, ducks, geese, pigeons, etc.) from different sources (wild birds, backyards, and commercial farms) is a suitable niche for persistence and perpetuation of AIV. Therefore, the partial or complete closure of LBM and effective cleaning and disinfection were effective procedures to temporarily stop poultry-to-human infections. Moreover, backyard birds were claimed to be the main source of human infections with H5N1 and H9N2 in Egypt, where women and children were more frequently infected due to slaughtering, defeathering, evisceration, or playing with infected birds [154,155]. Interestingly, few human infections were reported due to rearing or culling of farmed animals. Several serological surveillance studies have shown that zoonotic IAV infections are more frequent in workers of poultry and pig farms [156,157,158]. Moreover, AIV infection of humans (e.g., hunters) through the direct contact with wild birds is an additional risk factor [159,160].

## 5. Viral Determinants for Zoonotic Potential of IAV

The pathogenicity of IAV is a complex and multigenic trait, which is affected by several viral and host factors. Nowadays, the accessibility of sequencing data from a large number of viral strains together with the availability of different reverse genetics (RG) approaches for IAV [37,161,162,163], allow a better understanding of various host range and virulence determinants of IAV. Actually, intensive circulation of AIVs in animal reservoirs results occasionally in the emergence of genetic traits, which are at least sufficient to enable the virus to cross the animal-to-human transmission barrier. Furthermore, genetic changes improving viral fitness of zoonotic AIVs could been gained in the new (human) host [164]. Whether such genetic and phenotypic changes are part of the zoonotic potential needed for transmission or are part of the adaptation in the human host needs to be analyzed. For instance, different well-studied adaptive genetic changes in human AIV isolates (e.g., Q591K, E627K or D701N) were also detected in avian or other intermediate hosts, but to lesser extent [111,165,166,167]. To better assess the pandemic risk-potential of AIVs, a profound understanding for the implications of these (adaptive) changes on viral characteristics seems to be of great importance.

### 5.1. The Viral Polymerase Complex and NP Protein

Amino acid (aa) substitutions in the subunits of the RdRp have been reported to alter the replication, pathogenicity and host range of IAV in mammals and poultry [168]. The presence of specific aa at several positions in the PB2 subunit (627K, 526R/627K, 590S/591R, 701N, 253N/591K, 253N/291K, 158G, and 271A), were associated with an increase in viral polymerase activity and contributed to increased virus replication and pathogenicity in mammals [164,169,170,171]. Remarkably, a glutamic acid replacement at position 627 with lysine (E627K) in PB2 is an important host range determinant, which enhances efficient replication, transmissibility and pathogenicity of AIV in mammals [172,173]. One study reported the importance of position 627 as a determinant for the temperature sensitivity of viral RdRp and therefore for viral genome replication [174]. In general, human IAV prefer a temperature of approximately 33 °C to replicate in the upper respiratory tract (URT), whereas AIV replicate best at 41 °C in the intestinal tract. Contrary to AIV with 627E in the PB2, AIVs carrying a E627K substitution efficiently replicate at 33 °C in URT of mammals [175]. However, absence of 627K can be compensated by 701N to result in higher replication, transmissibility and virulence of AIV in mammals [176]. Both, the 627K and 701N seem to be human signature markers that have been found in most human isolates of H7N9 causing human infections and fatalities in China in 2013 [90]. Of note, the pandemic 2009 H1N1 virus is missing 627K and 701N without significant impact on virus replication and transmission in mammals [169,177], which is likely due to two compensatory residues, 590S and 591R [169].

In PB1, most avian IAV carry asparagine at position 375, whereas most human IAV including the pandemic 1918-H1N1, 1975-H2N2 and 1968-H3N2, contain a serine at this position [178]. Therefore, the aa residue 375 in PB1 might be critical for adaptation and virulence of avian IAV in mammals as a new species [178,179]. Retrospective analysis of the 1957 and 1968 pandemic strains suggested that avian PB1 in the genetic background of human IAV could promote efficient replication, transmission and virulence in mammals [180,181,182]. In addition, PB1-F2 contributes to higher pathogenicity of avian IAV in mammals and to bacterial secondary infection [13,14,15]. Unlike the truncated non-functional PB1-F2 protein in pandemic 2009 H1N1 viruses, PB1-F2 is suggested to exert an important role in the pathogenicity of the 1918, 1957 and 1968 pandemic strains as well as the HPAIV H5N1 viruses [15,183]. In PB1-F2, 66S increased the replication and virulence of IAV in mice and antagonized virus-induced cellular response. Interestingly, a S66N substitution attenuated the H5N1 and 1918/H1N1 viruses [184].

In the PA protein, 97I was shown to enhance the virulence of influenza virus A/Aquatic bird/Korea/W81/05 (H5N2) in mice rather than in chicken and to promote efficient replication in vitro in mammalian rather than avian cell lines [185]. In addition, specific PA residues (70V, 85I, 186S, 224S, 336M, 353R, 400P, 423M 476V, 552S and 630V) were shown to improve the viral polymerase activity of different IAVs in vitro and enhance virus replication and pathogenicity in mammals [164,186,187,188]. However, the PA-X protein was shown to rather act as a negative regulator of pathogenicity of 1918 H1N1 and HPAIV H5N1 in mice and avian host species, respectively [19,20,189]. The loss of PA-X expression (PA-X-null) improved viral replication, increased cell-death and host innate responses, and thereby virulence in mice and avian species [19,20,189]. To summarize, the PA-X modulates IAV virulence by impairing viral and host mRNA expression, thereby affecting virus propagation and host innate immune response.

Several studies have indicated an important role of the NP protein in viral replication and pathogenicity. It is now known that the cellular antiviral-acting Mx1 (mouse) or its human ortholog MxA interact with the viral NP and thereby inhibit IAV replication [190,191]. Therefore, the sensitivity of IAV to mammalian MxA is affected by the nature of the viral NP protein (e.g., avian strains of IAV are typically more sensitive to MxA than human strains) [192,193]. IAVs escape from MxA restriction by acquiring specific aa substitutions in the NP [194,195,196]. A novel deep mutational scanning approach identified several different sites in the viral NP that either increase the sensitivity or the resistance to MxA [197].

The classical nuclear import of vRNP is primarily regulated by adaptor importin-α proteins, which subsequently link the nuclear localization signals (NLSs) of imported vRNP molecules to importin-β and in turn mediate their nuclear localization [198,199]. The NP functions as a mediator between IAV-vRNP and the host cell karyopherins “importin-α.” The IAV-NP carries two main nuclear localization sequences (NLS). NLS1 ranges from aa positions 1–13 and NLS2 from aa positions 198–216 [200,201]. Similarly, importin-α interacts to different NLSs on viral polymerase subunits to mediate the nuclear import of vRNP such as NLS at aa residues 449–495 and 738–755 of PB2 [202], NLS at aa residues 187–211 of PB1 [203] and NLS at aa residues 124–139, E154, 186–247 of PA [204]. Interestingly, 319K (NP), 701N (PB2), and 627K (PB2) are important to adapt the binding of vRNP from influenza virus of avian origin to mammalian importin-α7 isoform [205,206,207,208]. However, IAVs with the avian signatures (627E and 701D) depend primarily on importin-α3 [207,208]. This switch in the usage of different importin-α isoforms from α3 to α7 following the acquisition of adaptive aa residues in NP and PB2 is crucial for efficient viral replication and higher polymerase activity in mammalian cells [207].

### 5.2. Viral Surface Glycoproteins (HA and NA)

The fourth segment of the IAV genome encodes for the hemagglutinin (HA), the major trimeric surface glycoprotein. It is synthesized as a single polypeptide precursor (HA0). To activate the HA fusion peptide that mediates fusion between the viral envelope and the host cell membrane, HA0 needs to be proteolytically cleaved into the two subunits, HA1 and HA2, that are held together via a disulphide bond [3,209,210].

The HA plays a key role in the replication and adaptation of IAV to new hosts [211] because it is responsible for the receptor binding of IAV to the host cell membrane. The affinity of HA to specific sialic acid (SA) residues is a main determinant of host range. AIV are known to preferentially attach to sialic acid bound to galactose via an α-2,3-linkage (α2,3-SA), which is predominant in the intestinal tract of birds [212,213], human lower respiratory tract (LRT, alveoli and bronchiolar cells junctioning bronchiole and alveoli) and conjunctival cells [214], whereas human IAVs prefer to bind to SA bound to galactose via an α2,6-linkage (α2,6-SA), which is predominant in the human URT including epithelial cells lining nasal cavity, paranasal sinuses, pharynx, larynx, trachea, and bronchi [214,215]. Importantly, both α2,3-SA and α2,6-SA have been detected in the URT of swine, as well as in quails giving rise to efficient replication of IAVs with affinity to human- and avian-type SA receptors [216,217].

The receptor binding residues of IAV are located in the HA head domain [218]. Several point mutations in the HA alter the SA receptor specificity, antigenicity, host range, replication efficiency and pathogenicity [219,220,221]. Moreover, cleavage of HA0 into the HA1 and HA2 subunits is needed to permit fusion of the viral envelope with the host endosomal membrane during virus entry to allow the viral genome to enter the cytoplasm. Thus, the distribution of HA-activating proteases in host cells is a major determinant for virus tropism and pathogenicity [61]. The cleavage site in the HA of HPAIVs (H5- and H7-subtypes) is composed of multiple basic aa residues. It can be cut by the ubiquitously expressed proprotein convertase “furin” and furin-like proteases, resulting in severe systemic infection in poultry and mammals [222,223]. Conversely, the HA0 proteins of LPAIVs possess a monobasic arginine (R) residue at their cleavage site and are only activated by extracellular proteases including tryptase Clara and bacterial proteases [224,225], which are restricted to respiratory and intestinal cells, only allowing localized infection [226].

To date, the molecular determinants of efficient replication, transmission and virulence for the seasonal IAV infection in mammals have been intensively studied. Several aa substitutions were found to be connected to changes in the receptor preference of HA in subtype specific manner such as N154S, N182K, Q192R, Q222L, S223N, G224S, Q226L, S227N, L129V + A134V, G139R + N182K, Q192R + S223N, Q222L + G224S (H5-subtype), E190D, D22G (H1-subtype), G225D (H1- and H9-subtypes), and G228S (H2-, H3-, H4-, H5-, and H9-subtypes) [164]. For instance, substitutions E190D and D225G were identified to change the binding preference of H1N1-HA toward human-like receptors leading to increased disease severity [227,228]. Otherwise, Q226L and G228S in the receptor binding domain of the H2- and H3-HA seem to be associated with increased affinity toward human-like receptors [228].

Furthermore, airborne transmission of AIV among humans is of special concern. In 2012, Imai et al. have shown that the introduction of 4 mutations (N158D/N224K/Q226L/T318I) into H5-HA via serial-passaging of the HPAIV H5N1 in ferrets changed the receptor binding preference towards α2,6-SA and enabled airborne virus transmission between ferrets [229]. Nevertheless, in a another study also concerning the adaptation of HPAIV H5N1 in ferrets, similar but not identical substitutions were found, leading to enhanced α2,6-SA binding enabling airborne virus transmission between ferrets [230]. Interestingly, serial passaging of an HPAIV H7N1 in ferrets also resulted in an airborne transmissible variant, with only one aa change in the stalk region of HA, but with four additional mutations in the internal proteins (PB2; NP, M1) [231].

Consistently, Q226L, located in the HA receptor binding domain, contributed to the adaptation of a H10N7 AIV to seals/mammals by improving a binding affinity to the human-like α2,6-SA receptor [146]. Recently, using a novel phylogenetic algorithm, Schmier et al. have predicted and experimentally confirmed the impact of three mutations (K153D, S223N and G272S) in the viral HA gene of Egyptian H5N1 AIV strains that are likely to play a critical role in changing the host cell receptor binding affinity towards mammals [232].

The neuraminidase (NA), encoded by the 6th segment of the IAV genome, forms a tetrameric spike protein with enzymatic activity. Since NA mediates the enzymatic removal of SA from the surface of infected host cells (Table 1), a balance between the strength of HA receptor-binding activity and the NA receptor-destroying activity is important for optimal infection, transmission and host adaptation of IAV [233,234]. Before its transmission to humans causing the Asian flu pandemic in 1957, the H2N2 virus possessed an NA with a preference to release progeny virions attached to avian α2,3-SA, whereas the HA of this virus already preferred mammalian α2,6-SA receptors. It is assumed that this N2-NA gradually adapted in the human population, infected by the progenitor of the “Asian flu” virus, and acquired the specificity to cleave human-type receptors [235]. The aa substitution I275V in the N2-NA was also shown to increase α2,6-SA receptor substrate specificity [236]. Furthermore, several studies have shown that specific deletions in the NA stalk domain—short-stalk NA—increase the virulence of AIVs in mice and poultry [237,238,239,240]. Nevertheless, short-stalk NA limits the transmission of pandemic H1N1 virus in ferrets [241].

### 5.3. Non-Structural Protein 1 (NS1)

The non-structural protein 1 (NS1) is small, multifunctional protein (mostly 230 aa/26 kDa) that is encoded by the eighth vRNA segment and translated from the unspliced viral mRNA of the NS segment. The NS1 protein exerts different activities in infected cells to promote efficient viral replication and virulence. NS1 interferes with type I IFN production as well as with IFN-inducing factors. In addition, it plays a positive regulatory role in RdRp activity and the nuclear export and translation of viral mRNAs [26,27,242]. In contrast, NS1 negatively regulates cellular pre-mRNA maturation and nuclear export, leading to preferred expression of viral genes [243]. Thereby, NS1 plays an important role as a regulator of viral replication, pathogenicity and host range through different protein-protein interactions [30,244,245,246]. For instance, by direct activation of viral mRNA translation through interactions with proteins involved in mRNA transport and translation (NXF1/TAP, Staufen, eIF4GI, and PABPI), NS1 potentiates viral replication [26,247]. Moreover, the ability of NS1 to antagonize the virus-induced antiviral response promotes viral replication [30]. Therefore, genetic drift by aa deletions, mutations and truncations that abolish or potentiate the ability of the NS1 to block the IFN production, can affect the overall yield and the pathogenicity of propagated virus [248]. For example, F103L and M106I substitutions in the NS1 increased virus replication and virulence of AIV in mammals [249]. In the NS1 of H5N1 viruses, the D92E substitution or the deletion of five aa residues (80–84) increased virulence in avian and mammalian animal models [250,251,252]. However, another naturally occurring five aa deletion (residues 191–195) in the NS1 of swine H5N1 viruses contributed to an inefficient ability of NS1 to control the IFN response and thereby reduced pathogenicity of these viruses in mice and chickens [253], as this deletion disrupted the essential binding of the NS1 to the splicing and polyadenylation factor CPSF-30 [253]. Furthermore, recent studies have shown that NS1-binding to CPSF-30 is also impaired by specific aa residues (108R, 125E, 189G) in the NS1 of the 2009 pandemic H1N1 virus (H1N1pdm09), resulting in reduced IFN antagonism [254]. Interestingly, the NS1 of contemporary H1N1pdm09-descendants has gradually restored its ability to antagonize the IFN response by acquiring 6 specific aa residues, 55K, 90I, 123V, 125D, 131E, and 205S [255].

Beside the IFN antagonistic function of the NS1 protein, the PDZ-ligand binding motif (PBM) at the C-terminal end of NS1, commonly ESEV, KSEV, and RSEV, was shown to enhance the pathogenicity of 1918 H1N1 and HPAIV H5N1(KSEV) in mice [28,256]. Differently, the insertion of the PDZ-binding domain into the truncated NS1 protein of H1N1pdm09 did not show a significant impact on viral replication or virulence in mammals [257]. The PBM of avian IAV is directly implicated in tight-junction (TJ) disintegration. IAVs with particular PBM (ESEV) can directly disrupt TJ by mediating the NS1 binding to the host proteins Dgl1 and Scribble that are essential for the formation of TJ. However, as the ESEV motif is limited to a subset of avian IAV strains, direct targeting of TJ by NS1 might not occur in all IAV infections [29,258].

In addition to gradual aa changes in the NS1 protein sequence, genetic shift or reassortment of the NS segment harbouring the NS1 gene extends the host range and tissue tropism, impairs the cellular immune response and alters the replication and pathogenicity of the reassortant virus in vitro and occasionally in vivo [27,244,245,246,259].

## 6. Control and Prevention of IAV

### 6.1. Vaccination against IAV

Vaccination is still the primary defence line and the most effective method to combat IAV infection [260] and would be needed to protect against spread of a zoonotic strain in the human population. Three types of influenza vaccines are currently licensed for human use: (1) inactivated whole- or split-virus; (2) live-attenuated virus; and (3) recombinant HA subunit vaccines. An influenza vaccine strain (IVS) is traditionally generated by co-inoculation of an IAV strain against which protective antibodies should be raised, together with the egg-adapted influenza virus A/Puerto Rico/8/1934 (H1N1, PR8) into embryonated eggs. The allantoic fluid is then harvested and highly replicating reassortant variants that provoke specific neutralizing and/or protective antibodies are identified by extensive genetic and antigenic screening [261,262]. To further improve the generation of IVS, reverse genetics (RG) systems have been employed to rescue recombinant IVS in a relatively short time overcoming the time consuming disadvantage of natural reassortment [161,263,264]. Using plasmid-based RG systems of IAV, the IVS possesses at least the immunogenic surface glycoproteins (HA and NA) of the circulating IAV in the genetic background of the PR8 strain [262,265].

Animal vaccination can reduce the chance of human infections with AIV with zoonotic potential. Nevertheless, AIV infections occur even in vaccinated birds and pigs and are frequently associated to vaccines, which demonstrated clear antigenic mismatches [266,267,268], or due to a novel introduction of an exotic strain or clade [110]. Consequently, the antigenic mismatch could result in an endemic situation due to delayed disease recognition and diagnosis, resulting in further spread of the infection among the sub-optimal vaccinated populations [269,270]. This is making it hard to select of a representative and genetically stable prepandemic candidate vaccine strain. Additionally, effective vaccination of animals against IAV can be influenced by several factors including, but not limited to, on-farm biosecurity measures [271,272], immune response of different bird species [272], immunosuppression, uncontrolled distribution and storage of the vaccines, and interference of maternal immunity with early vaccination of the offspring [273]. Rearing habits can also complicate the situation. For instance, mixing of various bird species, which is a common practice when raising backyard poultry in Egypt, can significantly hinder virus control. Kandeil and his colleagues indicated that vaccination is effective in backyard settings in Egypt. However, planning should consider the differential host responses and therefore vaccination effectiveness in the mixed species [272]. The low biosecurity conditions of backyard rearing and uncontrolled vaccination practices have probably fostered the generation of new antigenic variants of H5N1 in the Egyptian poultry sector over the last decade. Therefore, improving vaccination efficacy in domestic and backyard poultry must be considered as an important measure to prevent zoonotic transmission of AIV with pandemic risk potential.

Luckily, the human-to-human spread of H5N1 and H7N9 strains is yet not well established, which reduces the urgency to recommend a regular annual vaccination against both strains. Nevertheless, some countries maintain national stockpiles of pre-pandemic vaccines against AIVs (e.g., H5N1 and H7N9) to control infections if similar viruses should transmit from human-to-human [274,275]. An inherent uncertainty inevitably associated is the fact that possible pandemic strains may be antigenically distinct from the stockpiled AI vaccines. To this point, these vaccines stockpiles should be regularly updated in response to new (relevant) knowledge that may become available.

### 6.2. Antivirals against IAV

**Figure 7:**
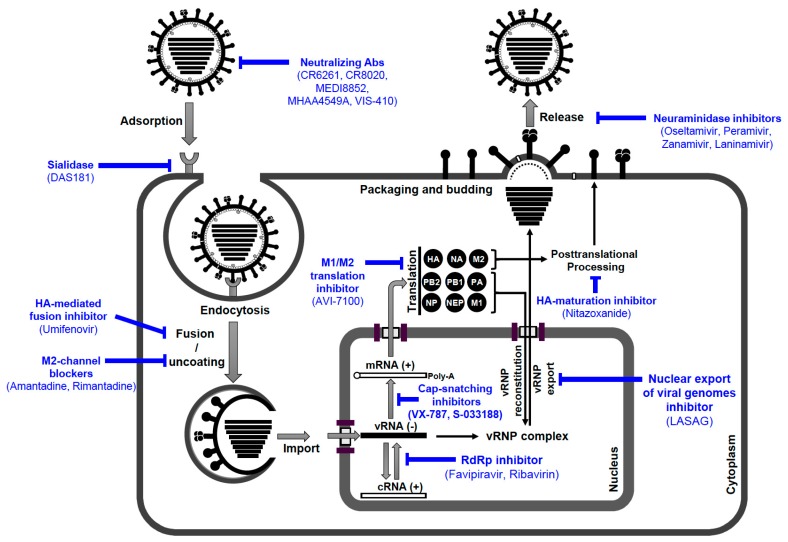
The targets of anti-influenza agents that are currently licensed or under clinically investigation. Before attachment of the influenza virus particle (IVP) to the host cell, specific neutralizing monoclonal antibodies (mAbs) against conserved domains in HA can prevent viral infection. Enzymatic destruction of the receptor determinant can further prevent IVP-binding to the target cells. After binding of the IVP to host cell sialic-acid receptors the viral life-cycle is continued by receptor-mediated endocytosis, HA-mediated fusion of the viral membrane with vesicular membrane, vRNP uncoating and release into the cytosol. The viral genome is then replicated/transcribed in the nucleus. After the viral mRNA has been translated into proteins some undergo post-translational processing in the cytosol or support genome replication in the nucleus. Newly formed vRNPs are exported from the nucleus and finally progeny virions are assembled and released by budding from the infected cell to infect new cells. These different processes are potential targets for the currently licensed antiviral drugs and others, which are in clinical trials including CR6261, CR8020, MEDI8852, MHAA4549A, VIS-410 (neutralizing Abs); DAS181 (sialidase); Umifenovir (fusion inhibitor); adamantanes (M2 channel blockers); Favipiravir and Ribavirin (RdRp inhibitors); VX-787 (PB2 cap-binding inhibitor); S-033188 (PA endonuclease inhibitor); AVI-7100 (inhibits M1/M2 mRNA-splicing); Nitazoxanide (HA maturation inhibitor); and Oseltamivir, Peramivir, Zanamivir, and Laninamivir (Neuraminidase inhibitors). In addition to its NF-κB inhibition effect, LASAG antagonizes the nuclear export of viral genomes and thereby blocks the assembly and release of mature influenza virus.

Should a zoonotic AIV against which vaccines have not yet been generated infect and spread in the human population antivirals will be urgently needed. The momentarily available antiviral medications to control IAV infection are optimally prescribed within 48 h of symptom onset for patients admitted to the hospital with influenza-like illness and individuals at high risk of developing influenza-related complications. Several essential preventive and therapeutic strategies could help to combat epidemic and pandemic infection with zoonotic strains, including the stockpiling of anti-influenza drugs [276]. Currently, many different approaches are being pursued in experimental, pre-clinical work, or are at different stages in clinical trials. Here, we focused on four classes of antiviral drugs targeting different viral or cellular factors, which are either licenced in the European Union and the USA, or are available in specific countries, for the treatment and prophylaxis of influenza virus infections [277,278]. They include (I) adamantanes, (II) neuraminidase inhibitors, (III) membrane fusion inhibitors (Russia, China), and (IV) RNA-dependent RNA polymerase inhibitors (Japan). Furthermore, several anti-influenza drugs are in late-phase clinical trials (Figure 7) [277,279]. The members of the predefined classes are commonly used separately for anti-influenza treatment. However, preclinical studies have demonstrated that combinations of antiviral agents with different mode-of-action might be beneficial [277].

#### 6.2.1. Adamantanes

The matrix protein 2 (M2) forms a homotetrameric integral membrane protein (97 aa). The M2 protein exerts an important proton channel activity that mediates the uncoating process through the acidification of the interior of the virion. This dissociates the M1 protein from the vRNPs mediating the release and the nuclear import of the vRNPs after HA-mediated fusion between viral and endosomal membrane [6]. Two orally active adamantane derivatives (amantadine, rimantadine) have been used for many years to control IAV infections by blocking the essential M2 channel function during the uncoating process of IAV, but they are not active against IBV [280,281]. However, resistance to both drugs has been vastly reported for the circulating seasonal IAVs and AIVs [281,282]. IAV resistance to adamantanes was associated with 6 aa mutations in the membrane-spanning region of the M2 protein (L26F, V27A, A30T/V, S31N, G34E, and L38F) [280,282]. It is noteworthy that S31N is the most common adamantanes-resistance marker in human (98%), avian (88%), and swine (77%) IAVs, followed by V27A [282,283,284]. Presently, the rapid emergence and the high resistance rates among seasonal and non-seasonal IAVs to adamantanes restricts their use for the treatment and prophylaxis of contemporary IAVs [283,285,286].

#### 6.2.2. Neuraminidase Inhibitors

The sialidase activity of NA is exerted by specific and highly conserved aa residues (active sites). These aa residues are differentiated into 8 catalytic- and 11 framework residues (Table 4) [287,288,289]. The catalytic residues interact directly with the substrate while the framework residues stabilize the catalytic residues. The catalytic site cleaves the α2-3-SA and α2-6-SA on the host cell membrane to release progeny virions from host cell. Therefore, inhibition of NA activity was thought to be an ideal strategy for antiviral drug development [288,290,291]. Neuraminidase inhibitors (NAIs), such as (1) orally administered oseltamivir (Tamiflu); (2) inhaled zanamivir (Relenza); and (3) intravenously applied peramivir (Rapivab), are FDA-approved anti-influenza drugs that act by inhibiting the sialidase activity of the IAV/IBV NA, thereby inhibiting the release of progeny virions from infected host cells [292]. Additionally, the inhaled Laninamivir (Inavir) is solely licensed in Japan for seasonal influenza viruses [293]. NAIs are currently the primary anti-influenza agents for the prevention and treatment of influenza virus infections, preferably within 48 h after onset of illness. [277,294]. Oseltamivir has been widely used because of its easy oral administration. However, zanamivir and peramivir are the drugs of choice when the effect of oseltamivir is limited due to oseltamivir-resistance. Due to differences in the chemical structures of the NAIs, resistance to oseltamivir may not necessarily confer resistance to the other two licensed NAIs, zanamivir and peramivir (Table 4) [295].

Regarding non-seasonal AIVs, the limited surveillance of the NAI sensitivity of circulating viruses, especially for the H5N1- and H7N9-strains, remains a problem. So far, only a few epidemiological/antiviral studies have described H5N1 and H7N9 subtypes with reduced oseltamivir sensitivity, found with low prevalence in both poultry and humans in different geographical localities [296,297,298,299,300]. Nevertheless, being the only globally approved influenza-specific antiviral, different countries are maintaining licensed NAIs stockpiles as an approach for pandemic preparedness.

The effect of antiviral drug resistance relies primarily on the viral replication fitness of the drug-resistant variant versus the drug-sensitive strain. This was shown in studies that investigated the relevance of specific aa residues (known to confer NAI resistance) for neuraminidase activity, viral replication fitness, transmissibility and virulence in vitro and in vivo. However the results were not consistent and were dependent on the subtypes studied (e.g., H1N1, H3N2, H5N1, H7N9) and their particular genetic differences [291,301,302,303,304]. Generally, it seems that the effectiveness of NAIs is reduced when viral resistance does not, or only modestly, compromise viral fitness (unaltered virulence, transmission, and growth rate) [305]. Therefore, in addition to antiviral resistance a timely and accurate monitoring of strain fitness is of crucial importance to global public health [295,301,305].

Despite that NAIs are presently effective in controlling infections by seasonal and zoonotic IAV strains, the emergence and global spread of NAIs-resistant IAV variants is a concern, especially in areas of high level of NAI use like Japan [300]. Consequently, new antiviral agents targeting viral proteins/functions are needed (Figure 7). Furthermore, as the high genetic variability of IAV allows them to quickly adapt to selective pressures, new approaches must be considered. The fact that all viruses depend on specific cellular functions has led to a paradigm change in the antiviral strategy, aiming at certain cellular functions/mechanisms that could be inhibited to impair viral replication without severe effects on the host [306,307,308].

#### 6.2.3. Membrane Fusion Inhibitors

As mentioned before, during the viral entry process, the pH decrease within the endosome leads to an irreversible conformational change in the HA2 subunit activating the fusion peptide, which interacts with the endosomal membrane to initiate the fusion process. Subsequently, the vRNPs are released into the cytoplasm and are then transported into the nucleus to initiate viral genome replication/transcription (Figure 7). Therefore, HA-mediated membrane fusion is targeted in anti-influenza virus approaches [277,278]. Umifenovir (Arbidol) is a broad-spectrum antiviral drug licensed in Russia and China for prophylaxis and treatment of respiratory infections including seasonal influenza [277]. Umifenovir blocks the fusion process via targeting the conserved HA stem domain and prevents the low pH-induced conformational rearrangements of the HA into its fusogenic state [277,313].

#### 6.2.4. RNA-Dependent RNA Polymerase (RdRp) Inhibitors

The RdRp complex comprises conserved and independently folded subdomains with defined functionalities in viral transcription and replication. To date, T-705 (Favipiravir), a purine pseudobase inhibitor of RdRp, is licensed in Japan for the treatment and prophylaxis of pandemic influenza A/H1N1 [277]. Ribavirin is another purine pseudobase that interferes with viral replication of influenza viruses. However, different studies have shown that ribavirin is only efficient against IAVs when administered in synergistic combinations with oseltamivir and amantadine [314].

### 6.3. Anti-Influenza Drugs in Late-Phase Clinical Trials

Due to the expansion of drug-resistance to M2-blockers and NAIs, new therapeutic strategies have been developed targeting the cellular components such as sialic acid receptors, posttranslational processing of the viral proteins, or essential proviral intracellular signaling cascades. DAS181 (Fludase) is a recombinant sialidase, which is designed to be applied by inhalation to restrict the binding of influenza viruses to the host cell by removing sialic acid receptors from glycan structures on the human airway epithelia [278].

Nitazoxanide, an orally active anti-parasitic drug, controls IAV infection by impairing the trafficking of the HA from the endoplasmic reticulum (ER) to the Golgi and inhibiting HA maturation by blocking HA terminal glycosylation. It also stimulates antiviral cellular innate immunity via interferon induction [277,278].

Currently, further inhibitors are under investigation in late-phase clinical trials: (i) VX-787, an orally active inhibitor of PB2; (ii) S-033188, an orally active PA inhibitor; and (iii) AVI-7100, intravenously active, small interfering antisense RNA constructs, targeting influenza M1/M2 translation [277,278,287]. More recently, Scheuch et al. showed that intranasally administered d,l-lysine-acetylsalicylate-glycine (LASAG), an anti-inflammatory derivative of Aspirin, resulted in a significantly faster alleviation of influenza symptoms, more rapid decline in viral shedding, and decreased risk of viral spread [306]. LASAG acts by inhibiting IKK-mediated NF-kB activity and blocking nuclear export of viral genomes [306]. The in vitro treatment with LASAG was not associated with the emergence of viral resistance [306,315].

Furthermore, five monoclonal antibodies that efficiently neutralize invading IAV are under evaluation in clinical trials. These mAbs target either the globular head or the highly conserved stem region of the viral HA, or the conserved ectodomain of the M2 (M2e); namely CR6261, CR8020, MEDI8852, MHAA4549A, and VIS-410 [277,278].

## 7. Conclusions

More than 50% of all human viral infections can be transmitted from animals. The One Health approach is based on the fact that animal, human and environmental healths are interrelated. A hundred years ago in 1918–1919, mankind was confronted with the first documented influenza pandemic of the 20th century, representing a devastating global occasion of influenza virus infections. Since then, influenza viruses showed a high genetic and antigenic diversity either by gradual acquisition of particular changes (antigenic drift) important for the adaptation to a new host or by monogenic/polygenic reassortment events (antigenic shift). This wide diversity of IAVs permitted them to adapt to different avian and mammalian host species including humans. In recent years, new AIVs emerged that have evolved independently to cross animal/human interface and have caused mild to fatal complications in human. Despite the massive screening for new antiviral drugs against influenza viruses, limited numbers of FDA-licensed or conditionally/regionally licensed anti-influenza drugs are available as a preventive strategy. Therefore, a better understanding of the genesis of newly emerging IAVs, improved surveillance efforts and intensified research to monitor and understand these foes are mandatory. Virtually, the One Health concept, a collaborative effort of different specialized experts across animal, human and environmental health to ameliorate animal and human health, must be implemented to fight against the high-consequence zoonotic AIVs.

## Figures and Tables

**Table 1:** The genome and functional proteins of influenza A virus (IAV).

Segment	vRNA(nt)	Viral Protein(s)	Protein		Molecules per Virion	Main Functions	Ref.
			(aa)	(kD)			
**1**	2341	PB2	759	80	30–60	(i) recognition and binding to the cellular mRNA cap sequence; (ii) RIG-I-mediated IFN-expression by binding to the mitochondrial antiviral signalling protein (MAVS)	[3,8,9]
		PB2-S1 *†	508	55	N/A	(i) inhibition of RIG-I-dependent interferon signaling pathway, (ii) interference with RdRp activity via competitive binding to PB1	[10]
**2**	2341	PB1	757	90	30–60	(i) captures of snatched Cap-structure to prime the viral mRNA transcription, (ii) transcribes vRNA into complementary RNA (cRNA) as template for further vRNA synthesis, (iii) initiates vRNA synthesis	[8]
		PB1-F2 †	87–90	10.5	N/A	(i) induces apoptosis, (ii) modulates host interferon response, (iii) modulates the susceptibility to secondary bacterial infection	[11,12,13,14,15,16]
		PB1-N40 †	718	≈80	N/A	maintains the balance between PB1 and PB1-F2 expression	[17,18]
**3**	2233	PA	716	83	30–60	RNA endonuclease to cleave small capped RNA structures to be used for viral mRNA synthesis	[3,8]
		PA-X †	252	29	N/A	modulation of the host response and viral virulence	[19,20,21]
		PA-N155 †	561	62	N/A	Promote viral replication and pathogenicity of IAV	[22,23]
		PA-N182 †	534	60	N/A		
**4**	1778	HA	566	77	500	(i) receptor binding; (ii) membrane fusion; (iii) major antigen	[3,8]
**5**	1565	NP	498	55	1000	(i) vRNA binding and protection; (ii) vRNA synthesis by vRNP complex; (iii) nuclear import of vRNP	[3,8]
**6**	1413	NA	454	56	100	(i) sialidase activity to release the virion progeny; (ii) help the viral particle to penetrate the mucus barrier of the respiratory tract to reach and infect the host cell	[3,8]
**7**	1027	M1	252	28	3000	(i) nuclear import and export of vRNPs; (ii) viral assembly, budding and morphogenesis	[3,8,24]
		M2 *	97	15	20–60	(i) Ion channel activity; (ii) uncoating process	[3,6,8]
		M3 *†	9	N/A	N/A	N/A	[25]
		M4 *†	54	N/A	N/A	N/A	
		M42 *†	99	N/A	N/A	Functionally complements M2	
**8**	890	NS1	230	26	N/A	(i) vRNP entry by hijacking importin-α; (ii) antagonize cellular antiviral responses including interferons; (iii) support viral mRNA splicing, maturation and translation; (iv) inhibits cellular mRNA maturation and translation	[8,26,27,28,29,30]
		NEP/NS2 *	121	14	130–200	(i) important for vRNP nuclear export; (ii) regulates vRNA transcription/replication	[31,32,33,34]
		NS3 *†	187	20	0	N/A	[35]

N/A means unknown; † refers to auxiliary viral proteins; * refers to viral proteins translated from spliced mRNA; aa: amino acids; kD: kilo Dalton.

**Table 2:** Host range of different hemagglutinin (H1–H18) and neuraminidase (N1–N9) subtypes [43,44,54,68,69,70].

HA-Subtype	NA-Subtype	Human	Swine	Equine	Domestic Poultry	Waterfowl Shorebirds	Sea Mammals (Seal/Whale)	Bat
H1	N1	H1/N1	H1/N1		H1/N1	H1/N1	H1	
H2	N2	H2/N2	H2/N2		H2/N2	H2/N2	N2	
H3	N3	H3/N3	H3	H3	H3/N3	H3/N3	H3/N3	
H4	N4	N4	H4		H4/N4	H4/N4	H4	
H5	N5	H5	H5		H5/N5	H5/N5	N5	
H6	N6	H6/N6	H6/N6		H6/N6	H6/N6		
H7	N7	H7/N7		H7/N7	H7/N7	H7/N7	H7/N7	
H8	N8	N8		N8	H8/N8	H8/N8		
H9	H9	H9/H9	H9		H9/N9	H9/N9	N9	
H10	N10	H10			H10	H10	H10	N10
H11	N11				H11	H11		N11
H12					H12	H12		
H13					H13	H13	H13	
H14					H14	H14		
H15					H15	H15		
H16					H16	H16		
H17								H17
H18								H18

**Table 3:** Confirmed human infections/fatalities with LPAIV- or HPAIV H7N9 per wave.

Wave Number	Period	Phenotype	Cases	Fatalities	Fatality Rate
1	February 2013–September 2013	LPAIV	134	45	34%
2	October 2013–September 2014	LPAIV	306	131	43%
3	October 2014–September 2015	LPAIV	219	102	47%
4	October 2015–September 2016	LPAIV	114	47	41%
5	October 2016–September 2017	LPAIV/HPAIV	848	295	35%
6	Since October 2017	LPAIV/HPAIV	4	3	75%

**Table 4:** Active site residues for all NA-subtypes and genetic resistance markers in viral NA proteins of highlighted non-seasonal human IAVs [288,290,291,296,300,304,308,309,310,311,312].

NA Active Site (N2 Numbering)		Influenza Virus Type/Subtype	Resistance Marker (N2 Numbering)/Location within NA Active Site								
Catalytic Residues (CR)	Framework Residues (FWR)		Oseltamivir			Zanamivir		Peramivir		Laninamivir	
			Amino Acid (aa)		Site	Amino Acid (aa)	Site	Amino Acid (aa)	Site	Amino Acid (aa)	Site
118R 151D 152R 224R 276E 292R 371R 406Y	119E 156R 178W 179S 198D 222I 227E 274H 277E 294N 425E	Non-seasonal A/H5N1 ^†^ and A/H7N9	A/H5N1	V116A	-	V116A	-	**E119D/G**	FWR		
				E119A/D	FWR	**E119A/D/G**	FWR	**H274Y**	FWR		
				Q136L	-	**Q136L**	-	N294S	FWR		
				D198G	FWR	D198G	FWR	**E119A/D/G + H274Y**	FWR		
				I222M	FWR	N294S	FWR	**I222M/V + H274Y**	FWR		
				S246N	-	K432T	-				
				**H274Y**	FWR	E119A/D/G + H274Y	FWR				
				**N294S**	FWR						
				I117V + I314V	-						
				**E119A/D/G + H274Y**	FWR						
				I222L + S246N	FWR/-						
				**I222M/V + H274Y**	FWR						
			A/H7N9	E119A/D	FWR	**E119A/D/G**	FWR	E119A/G	FWR	E119A	FWR
				I222K/R	FWR	**Q136K**	-	**E119D**	FWR	**E119D/G**	FWR
				T247P	-	I222K	FWR	**Q136K**	-	**Q136K**	-
				H274Y	FWR	T247P	-	I222K	FWR	R152K	CR
				E276D	CR	**E276D**	CR	E276D	CR	I222K/R	FWR
				**R292K**	CR	**R292K**	CR	**R292K**	CR	E276D	CR
				R371K	CR	N294S	FWR	R371K	CR	R292K	CR
				E119V + E222V	FWR	R371K	CR			R371K	CR

Bold refers to aa substitutions that confer high reduced inhibition (HRI) of NAI. Non-bold aa substitutions were shown to be associated with medium reduced inhibition.
